# Comparative transcriptome analysis reveals potential regulatory genes involved in the development and strength formation of maize stalks

**DOI:** 10.1186/s12870-025-06276-5

**Published:** 2025-03-01

**Authors:** Senan Cheng, Youhui Qi, Dusheng Lu, Yancui Wang, Xitong Xu, Deyun Zhu, Dijie Ma, Shuyun Wang, Cuixia Chen

**Affiliations:** College of Agronomy, Shandong Agricultural University, Tai’an, 271018 China

**Keywords:** Maize, Stalk development, Stalk strength, Transcriptome, Regulatory genes

## Abstract

**Background:**

Stalk strength is a critical trait in maize that influences plant architecture, lodging resistance and grain yield. The developmental stage of maize, spanning from the vegetative stage to the reproductive stage, is critical for determining stalk strength. However, the dynamics of the genetic control of this trait remains unclear.

**Results:**

Here, we report a temporal resolution study of the maize stalk transcriptome in one tropical line and one non-stiff-stalk line using 53 transcriptomes collected covering V7 (seventh leaf stage) through silking stage. The time-course transcriptomes were categorized into four phases corresponding to stalk early development, stalk early elongation, stalk late elongation, and stalk maturation. Fuzzy c-means clustering and Gene Ontology (GO) analyses elucidated the chronological sequence of events that occur at four phases of stalk development. Gene Ontology analysis suggests that active cell division occurs in the stalk during Phase I. During Phase II, processes such as cell wall extension, lignin deposition, and vascular cell development are active. In Phase III, lignin metabolic process, secondary cell wall biogenesis, xylan biosynthesis process, cell wall biogenesis, and polysaccharide biosynthetic process contribute to cell wall strengthening. Defense responses, abiotic stresses, and transport of necessary nutrients or substances are active engaged during Phase IV. Kyoto Encyclopedia of Genes and Genomes (KEGG) analysis showed that the two maize lines presented significant gene expression differences in the phenylpropanoid biosynthesis pathway and the flavonoid biosynthesis pathway. Certain differentially expressed genes (DEGs) encoding transcription factors, especially those in the NAC and MYB families, may be involved in stalk development. In addition, six potential regulatory genes associated with stalk strength were identified through weighted gene co-expression network analysis (WGCNA).

**Conclusion:**

The data set provides a high temporal-resolution atlas of gene expression during maize stalk development. These phase-specific genes, differentially expressed genes, and potential regulatory genes reported in this study provide important resources for further studies to elucidate the genetic control of stalk development and stalk strength formation in maize.

**Supplementary Information:**

The online version contains supplementary material available at 10.1186/s12870-025-06276-5.

## Background

Maize (*Zea mays* L.) is a globally important crop for food, feed, and energy production. Improving the yield and quality of maize grain has been a long-term goal for maize breeders, and stalk strength is one of the key factors influencing maize yield and quality [1, 2]. Stalk development significantly influences the growth rate, final height of the plant, and its resistance to lodging, thereby affecting crop yield and the efficiency of mechanized harvesting. During growth, maize stalk strength increases following an S-shaped curve [3], and is modulated by complex biological processes that determine epidermal thickness and deposition of lignin and cellulose in the cell wall [4, 5]. The regulatory networks controlling maize stalk development and stalk strength are still not fully understood; therefore, it is helpful to examine the temporal resolution dynamic transcriptome of individual internodes to identify potential regulatory factors involved. Indeed, many recent studies have used next-generation sequencing technology to investigate transcriptomic differences between different internodes and different developmental stages of maize stalks. An example of such studies is that the expression of genes associated with cell wall metabolism was greater in the Iowa Stiff Stalk (SS) line than in non-Stiff Stalk (NSS) line in the third internode at V9 stage and tasseling stage [6]. Recent transcriptomic studies have also identified several key genetic modules and genes that underpin the maintenance, extension, and division of internodes during stalk elongation and maturation [7]. A detailed comparison of the transcriptomes of maize stalks across various developmental stages between maize lines of contradistinctive stalk strength is of practical value.

Maize stalk lodging is a pressing issue in farming, and high stalk strength can significantly reduce lodging [8]. Maize stalk strength is determined by the morphological characteristics, anatomical structure, and carbohydrate polymer composition of the stalk [9, 10]. Some of the genes that modulate these aspects have been confirmed or proposed to influence stalk strength. For example, the chitinase-like1 protein, a protein that is highly expressed in elongated internodes, increases stalk strength when over-expressed [11]; the NAC transcription factors *ZmNST3* and *ZmNST4*, which are specifically expressed in secondary wall-forming cells, control the thickness of the secondary wall of the stalk (and thus the stalk strength), although genes regulated by these factors are not yet known [12]; and the disruption of *stiff1*, encoding an F-box domain protein, has a strong effect on enhancing stalk strength [1]. However, stalk strength is determined by multiple factors in likely dynamic and interweaving processes during stalk development, and it would be helpful to examine the gene expression profiles at each stage of plant development. In this study, nine developmental stages (V7 through silking) were selected for transcriptome analysis of two maize inbred lines with clearly different stalk strengths (CML323, strong stalk; W22, weak stalk). By examining the dynamic expression patterns of expressed genes at the large span of development stages, and by analyzing the differences in gene expression between the two inbred lines and their implication in physiological pathways, this study aims to provide expanded information for a better understanding of the genetic basis of stalk strength.

## Methods

### Plant materials, stalk strength measurement, and RNA sequencing

The CML323 and W22 inbred lines were planted at the experimental station of Shandong Agricultural University, Tai’an, China (36.18 °N, 117.13 °E). Each inbred line was planted in two rows (0.4 m between rows) and 0.2 m between plants in a 20 m × 1.2 m plot. Rind penetrometer resistance was measured at the middle of the third above ground from the seventh leaf stage to silking using IMADA digital force gauge (ZTS-50 N, IMADA Company, Japan), and at least three plants were measured at each time point. Each plant was measured three times, and the average was recorded. The rind of the third internode above ground (including the part of the pith that is connected to the rind) was immediately collected by manual dissection and frozen immediately in liquid nitrogen during the development stages of seventh leaf stage (V7), eighth leaf stage (V8), ninth leaf stage (V9), tenth leaf stage (V10), twelfth leaf stage (V12), fourteenth leaf stage (V14), fifteenth leaf stage (V15), tasseling stage (VT), and silking stage (R1), then stored at -80 °C before processing. Each sample contained tissues from at least two plants, and there were 3 replicates (called a set of samples hereafter) for each time point for each line (there were 2 replicates for W22 line at VT). Total RNA was extracted using TRIzol reagent (Vazyme). The cDNA libraries were sequenced on a NovaSeq platform (Illumina) to generate 150 nucleotide paired-end reads.

### RNA-seq data analysis

The B73 reference genome [13] was downloaded from MaizeGDB (https://download.maizegdb.org/Zm-B73-REFERENCE-GRAMENE-4.0/). The RNA-seq data was quality-controlled using fastp (v0.21.0) software with default parameters [14]. Subsequently, the filtered data were mapped to the B73 reference transcriptome using Salmon (v1.5.2) with mapping-based mode [15], and transcripts per million (TPM) values were calculated. The Pearson correlation coefficient among biological replicates was calculated after log_2_(TPM + 1) transformation (normalization). Hierarchical clustering was performed to generate co-expression clusters and principal component analysis (PCA) was performed to classify principal components, both using the R software (v3.6.1) [16].

Genes with TPM > 1 in at least two sets of samples were considered expressed. Expressed genes with a coefficient of variation (CV) ≤ 0.4 were deemed low variation genes and removed, and the remaining expressed genes were called *dynamically expressed genes*. These genes were clustered using the Mfuzz package [17] in R software v3.6.1.

### Differential expression analysis

Differential expression analysis of the two maize lines at each time point was performed using the DESeq2 package (v1.42.0) [18] in R software v4.3.2. Genes with corrected p value < 0.05 and absolute values of fold change > 2 were considered to be differentially expressed genes (DEGs).

### Functional enrichment and gene co-expression network analyses

The clusterProfiler package (v4.10.0) [19] in R software v4.3.2 was used for Gene Ontology (GO) enrichment analysis and Kyoto Encyclopedia of Genes and Genomes (KEGG) pathway enrichment analysis. GO terms with a corrected p value < 0.05 were considered to be significantly enriched. KEGG pathways with a corrected p value < 0.05 were considered to be significantly enriched.

Weighted gene co-expression network analysis (WGCNA) of the dynamically expressed genes was performed with R software (v3.6.1) using the WGCNA package [20]. The soft-thresholding power value β was calculated by the pick Soft Threshold function of the WGCNA package. Optimal soft-thresholding power value was the powerEstimate (power = 8, R^2^ = 0.87). By applying hierarchical clustering algorithm implemented in the WGCNA package, genes were clustered into modules using the one-step automated blockwiseModules function. The kME of each gene was obtained using the signed key module membership values (kME) function, and a gene with kME > 0.8 was considered to be the key regulatory gene in the blue module. The gene co-expression network (blue module) was visualized using cytoscape v3.8.0 (http://cytoscape.org/) [21].

### qRT‒PCR analysis

An aliquot of the total RNA used for constructing cDNA libraries for RNA-seq was used for independent qRT‒PCR analysis. Total RNA was reverse transcribed to cDNA using a HiScript II RT SuperMix (Vazyme, Nanjing, China). Quantitative PCR was performed with the ChamQ SYBR qPCR Master Mix (Vazyme, Nanjing, China) on an ABI QuantStudio 6 Flex Real-Time PCR system (Applied Biosystems, CA, USA). The maize *cullin2* gene (*Zm00001d024855*) was used as an internal control for the normalization of gene expression [22]. Relative expression levels were calculated using the comparative CT (2^−∆∆Ct^) method. All primer sequences are listed in Table S10.

## Results

### Generation of sequential transcriptome data of the two maize inbred lines

**Fig. 1:**
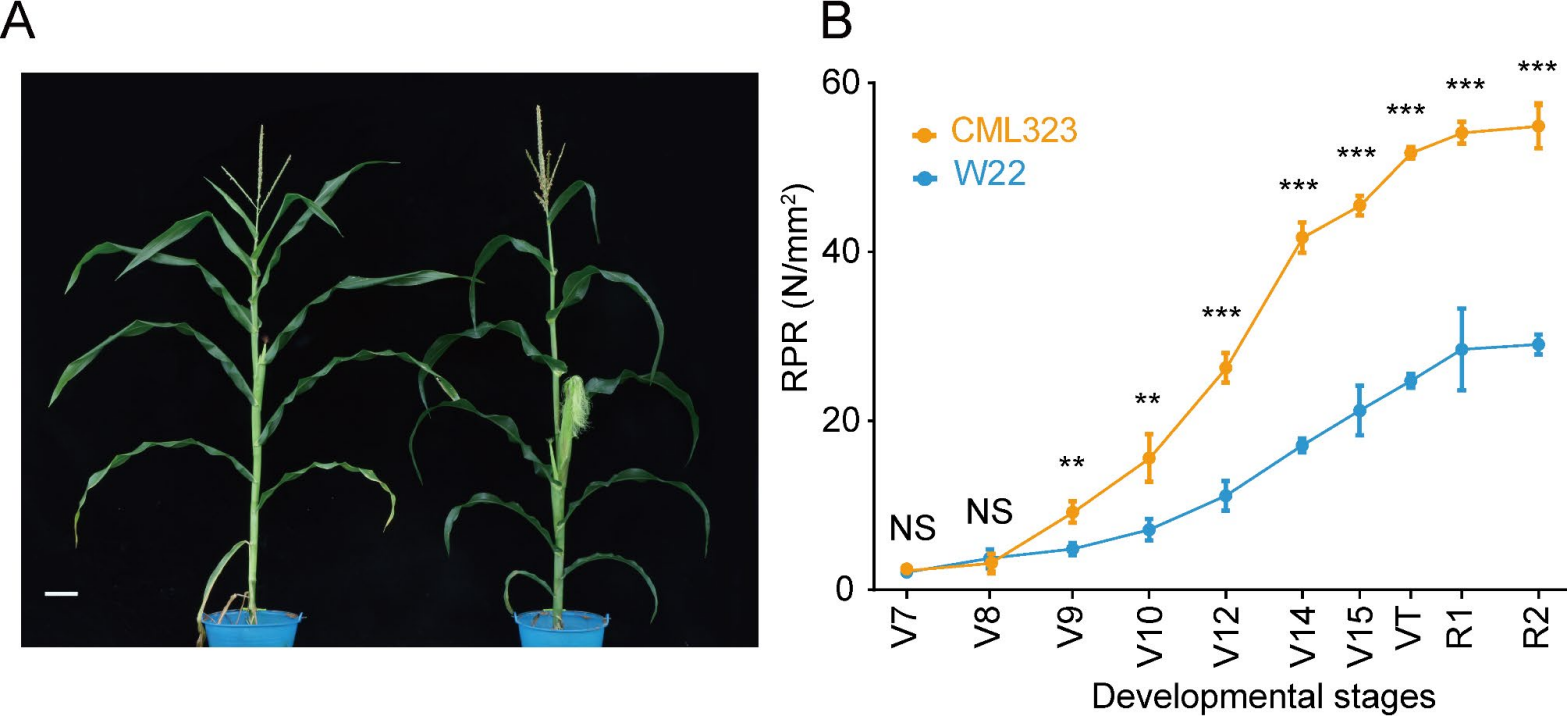
Stalk strength phenotypes of maize inbred lines CML323 and W22. (**A**) Plants of CML323 (left) and W22 (right) at R1 stage; bar = 10 cm. (**B**) Rind penetrometer resistance evolution in CML323 (*n* = 3 at each point) and W22 (*n* = 3 at each point); mean ± SD. VT, tasseling stage; R1, silking stage; R2, blister stage; RPR, rind penetrometer resistance. two-tailed Student’s t-test was used to determine P values. **P < 0.01; ***P < 0.001; NS, not significant

To understand the transcriptional control of maize stalk development and stalk strength formation at each developmental stage, we collected aboveground third stalk rind from CML323 and W22 from seventh leaf stage (V7) through silking stage (R1). The two inbred lines presented S-shaped curves for rind penetrometer resistance (RPR) from V7 to R2 (blister stage), reaching a plateau after the silking stage (Fig. 1B). The most significant increase in RPR for CML323 occurred from V10 to V14. The RPR of the two lines were similar at V7 and V8, but the RPR of CML323 increased more rapidly after V8, and the values of the two inbred lines were significantly different from those of V9. This finding indicates that the sampling time window included the critical stages of maize stalk development and stalk strength formation.

We extracted RNA from the rind of the third stalk above ground in the two inbred lines at each of the 9 development stages (V7 through R1), and a total of 53 transcriptome libraries were sequenced. RNA-Seq data were mapped to the B73 reference transcriptome (B73 v4) using Salmon (v1.5.2). On average, there were 50.91 million clean reads were obtained per sample after filtering (Table S1). The gene expression level was calculated as transcripts per million (TPM). Biological replicates had an average R^2^ of 0.98 (ranging from 0.93 to 0.99). Applying our set of criteria, we identified a total of 16,496 dynamically expressed genes (TPM > 1 in at least two sets of 18 samples, CV > 0.4), including 1,048 transcription factor genes, for the two inbred lines.

### Development phase progression is manifested by dynamic gene expression

**Fig. 2:**
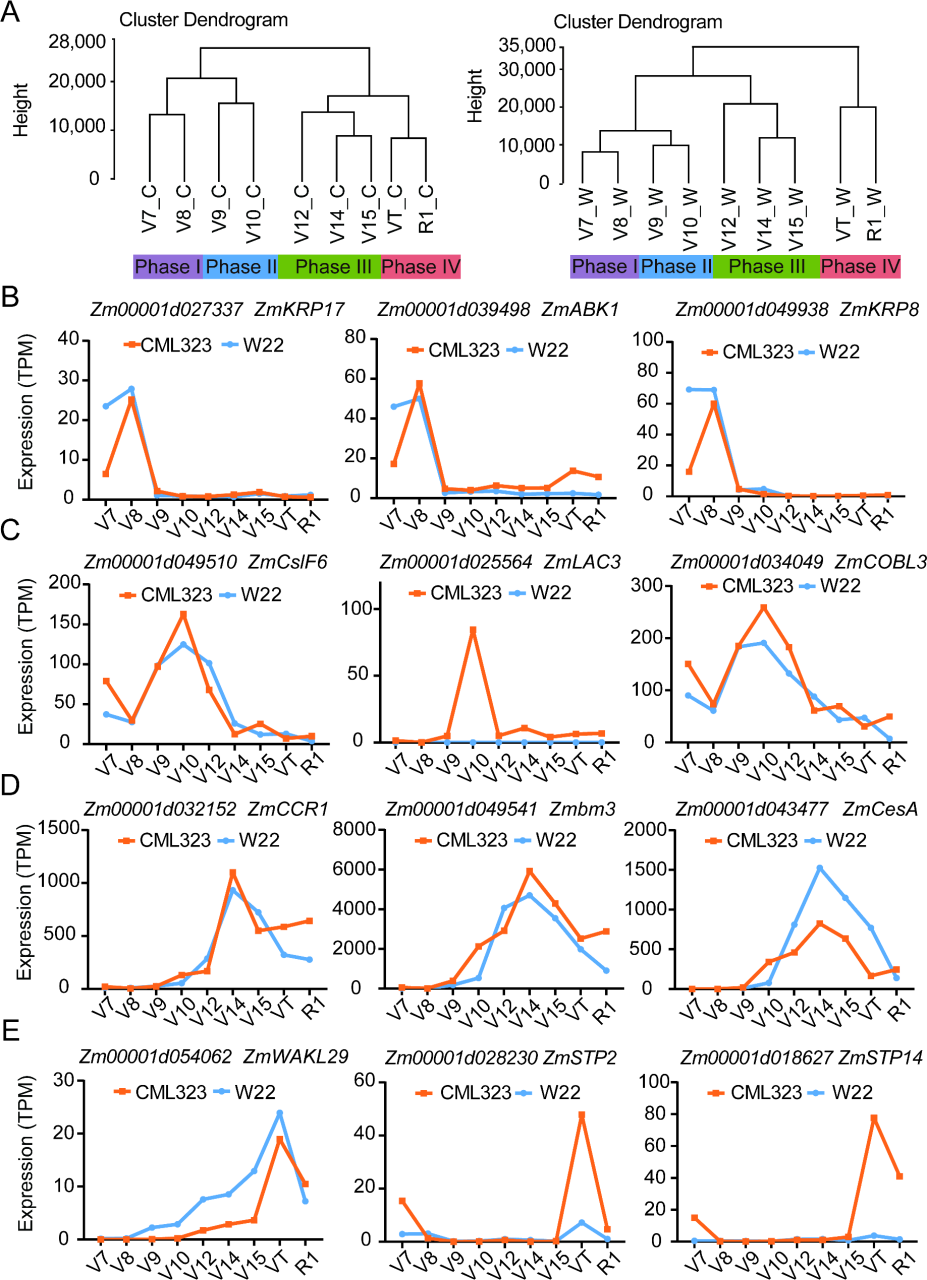
Hierarchical clustering of dynamically expressed genes spanning V7 to silking (R1) and representative genes that showed temporal expression pattern at the four development phases. (**A**) Cluster dendrograms of the transcriptomes of CML323 (left panel) and W22 (right panel). C, CML323; W, W22. These selected genes were mainly expressed in phases I (**B**), II (**C**), III (**D**), and IV (**E**) correspondingly

Through hierarchical clustering, the transcriptomes of CML323 and W22 could be divided into four groups, each corresponding to a specific temporal expression pattern, including Phase I (V7 + V8), Phase II (V9 + V10), Phase III (V12 + V14 + V15), and Phase IV (VT + R1) in this study (Fig. 2A). Principal component analysis produced three principal components in each line that explained 61% of the total sample variance in the transcriptome data in CML323 and 69.5% of the total sample variance in the transcriptome data in W22 (Fig. S1).

Phase I was a relatively early phase of stalk development. Conceivably genes related to cell division and intracellular transport could have been dominantly expressed in this phase. Figure 2B shows examples of these genes: *Zm00001d027337* encodes a kinesin-carrying malectin domain protein, presumably playing a role in cell division [23]; *Zm00001d039498*, a homolog of *AT2G25880* that encodes a member of the Ser/Thr protein kinase family whose transcripts are abundant in tissues rich in dividing cells but are low or absent in fully expanded tissues [24]; and *Zm00001d049938*, encoding a kinesin-related protein (KRP), which is presumably an inhibitor of cyclin and negatively regulates cell numbers and cell division [25]. Phase II annotates the period of fast elongation of the stalk, involving cell differentiation and cell wall reinforcement. Examples of actively transcribed genes in this phase are shown in Fig. 2C. *Zm00001d049510* is an ortholog of the *CslF6* gene in rice, and is involved in primary cell wall formation by regulating mixed-linkage glucan biosynthesis [26]; *Zm00001d034049* encodes a COBRA-like protein essential for proper cellulose deposition and cell wall formation during maize stalk phloem development [27]; *Zm00001d025564* (*ZmLAC3*), encoding a copper-containing laccase, controls the lignin content and RPR [28]. Notably, the expression level of *ZmLAC3* was very low in W22 throughout the studied development stages, which might be one of the factors accountable for the weak stalk phenotype of W22. Phase III (Fig. 2D) represents the period of rapid stalk development of stalk rinds and vascular bundles, and the formation of stalk strength. Lignin and cellulose production and organization play important roles in secondary cell wall thickening of cells in xylem, phloem, and sclerenchyma. *Zm00001d032152* (*ZmCCR1*) plays a role in regulating the composition of lignin monomers in maize, in particular the formation of H-type lignin [29]; *Zm00001d049541* affects maize resistance to lodging by influencing the ratio of G/S lignin [30]; *Zm00001d043477* encodes a cellulose synthase and affects cell wall structure and plant brittleness by regulating cellulose synthesis in maize [31]. Phase IV (Fig. 2E) denotes the transition from vegetative to reproductive growth, during which the elongation of the stalk slows down along with further strengthening of the stalk. For example, *Zm00001d054062* (*wakl29*) encodes a putative wall-associated receptor protein kinase (WAK) family protein. WAK and WAK-like proteins have known roles in normal plant development and disease defense responses [32–35]; *Zm00001d028230* (*ZmSTP2*) encodes a sugar transport protein, play an important role in the resistance of maize to *C. heterostrophus*, *C. carbonum*, and *S. turcica* [36]; *Zm00001d018627* (*ZmSTP14*) encodes a monosaccharide transporter, and is expressed in central starchy endosperm and embryo at the later filling and maturity stages [37]. These results indicate that these highly expressed genes in this late phase might be associated with biotic and abiotic stress responses and the transport of necessary nutrients or substances. Although our focus in this study was the development of the stalk, the division of growth phases might be useful as a type of guideline when examining physiological events in other tissues or the whole plant during subtle development progressions.

### Transcript clusters in stalks spanning the four phases

**Fig. 3:**
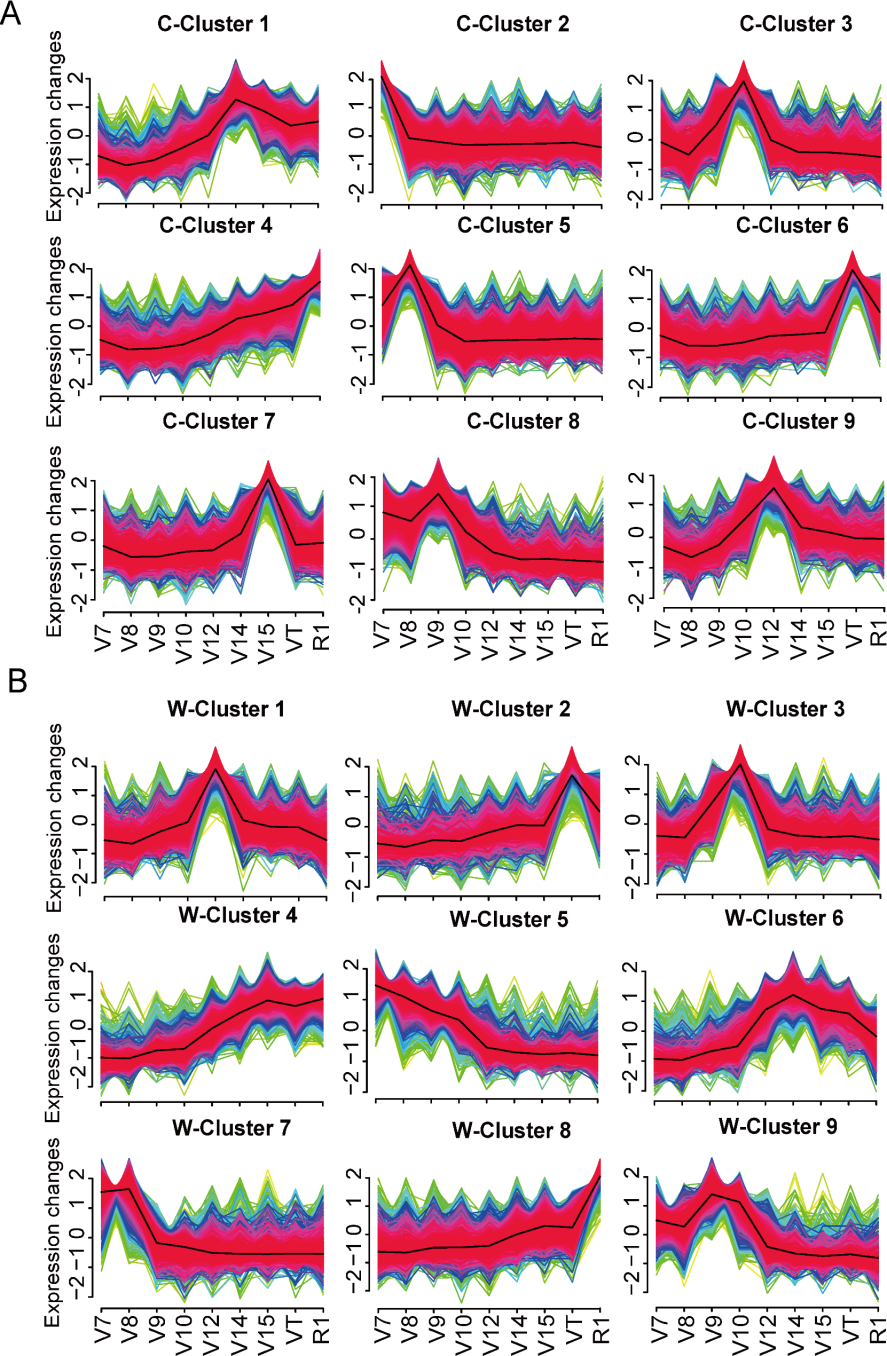
Clustering of dynamically expressed genes. (**A**) Gene expression clusters in CML323. (**B**) Gene expression clusters in W22. Fuzzy c-means clustering shows the dynamic expression profile of the 9 clusters throughout the studied development stages in each line. C, CML323; W, W22

To dissect transcriptional progression during maize stalk development, the expression patterns of the 16,496 dynamically expressed genes in CML323 and W22 were clustered into nine co-expression clusters for each line using the fuzzy c-means clustering algorithm (Fig. 3A-B, and Table S2, S3). To gain further insight into the functional transitions during stalk development, we conducted Gene Ontology (GO) enrichment analysis on nine co-expression clusters of CML323 and nine co-expression clusters of W22 to annotate the potential functions of these clusters (Fig. S2, S3).

In CML323, clusters 2 and 5 contained 1421 and 3824 genes, respectively, that were actively transcribed in Phase I (Fig. 3A). GO enrichment analysis suggested that processes of photosynthesis and the generation of precursor metabolites and energy were enriched in cluster 2 (Fig. S2B), whereas DNA replication and mitotic processes were enriched in cluster 5 (Fig. S2E). In W22, cluster 7 contained 2798 genes and cluster 5 had 2283 actively transcribed genes at phase I (Fig. 3B). GO term enrichment analysis suggested that the regulation of DNA replication and cell cycle processes were enriched in cluster 7 (Fig. S3G), and phospholipid metabolic process, plant-type cell wall organization, and microtubule organization were enriched in cluster 5 (Fig. S3E). These enriched biological processes suggest the active cell division in stalk during Phase I and the active physiological processes were similar in the two inbred lines.

Clusters 3 and 8 of CML323 showed peak gene expression at Phase II, a time when the fast elongation of maize stalks started (Fig. 3A). Processes including photosynthesis, generation of precursor metabolites and energy, cell wall biogenesis, and lignin biosynthesis were mostly enriched in cluster 3 (1441 genes; Fig. S2C), and cell wall tissue development, brassinosteroid (BR) mediated signaling pathway, external encapsulating structure organization, and other processes were enriched in cluster 8 (1949 genes; Fig. S2H). Similarly, for inbred line W22, cluster 3 (1544 genes) and cluster 9 (1625 genes) showed peak gene expression in Phase II (Fig. 3B). Processes including plant-type cell wall organization or biogenesis, cuticle biosynthesis process, water transport, lignin biosynthesis process, and regulation of secondary cell wall biogenesis were enriched in cluster 3 (Fig. S3C), while transmembrane receptor protein serine/threonine kinase signaling pathway, enzyme-linked receptor protein signaling pathway, cell surface receptor signaling pathway, and other processes were enriched in cluster 9 (Fig. S3I). These clusters are involved in processes required for cell division, elongation, and deposition, such as photosynthesis, cell wall organization and biogenesis, lignin biosynthesis, BR-mediated signaling pathway, and regulation of secondary cell wall biogenesis. BR are essential plant hormones whose biosynthesis is regulated by a rate-limiting enzyme encoded by *DWF4* and influenced by two bHLH transcription factors (TFs), *PIF4* and *TCP1* [38, 39]. *BES1/BZR1* homolog 3 (*BEH3*) antagonizes other *BES/BZR1* by competing for binding to the BR response element, which in turn regulates vascular robustness [40]. These results were in line with processes associated with cell wall extension, lignin deposition, and vascular cell development during stalk elongation.

There were 1762, 1275, and 1070 genes in clusters 1, 7, and 9, respectively, of CML323 that were preferentially expressed during Phase III (Fig. 3A). The enriched processes in cluster 1 were phenylpropanoid biosynthesis, lignin metabolic, secondary metabolite biosynthesis and monoatomic anion transport (Fig. S2A). And those in cluster 7 were response to salicylic acid, response to chitin, regulation of cellular amino acid metabolism process, and regulation of hormone metabolism process (Fig. S2G). The enriched processes in cluster 9 were secondary cell wall biogenesis, carbohydrate biosynthesis, and xylan biosynthesis (Fig. S2I). The 1210 genes in cluster 1 and 1809 genes in cluster 6 for W22 were preferentially expressed during Phase III (Fig. 3B). Processes like response to fungi and chitin, cell wall biogenesis, and response to nitrogen compound were enriched in cluster 1 (Fig. S3A), and processes such as cell wall biogenesis, polysaccharide biosynthetic process, phenylpropanoid biosynthetic process were enriched in cluster 6 (Fig. S3F), which was similar to cluster 1 in CML323. These clusters are involved in processes required for cell wall strengthening, such as lignin metabolic process, secondary cell wall biogenesis, xylan biosynthesis process, cell wall biogenesis, and polysaccharide biosynthetic process.

The cluster 6 of CML323 (1525 genes) had high expression level at the VT stage of Phase IV (Fig. 3A), and GO terms including secondary metabolic process, secondary metabolite biosynthesis process, inorganic ion transmembrane transport, and response to fungi were enriched (Fig. S2F). The expression of cluster 2 (882 genes) and cluster 8 (1622 genes) in W22 were elevated in Phase IV (VT and R1; Fig. 3B). Genes in clusters 2 and 8 of W22 correlated mainly to stress response processes such as response to reactive oxygen species, transmembrane transport, response to heat, response to toxic substances (Fig. S3B and S3H). These results were consistent with coping with defense responses, abiotic stresses, and transport of necessary nutrients or substances during the later stages of plant development.

In addition, expression of the 1888 genes in cluster 4 of CML323 and the 2336 genes in cluster 4 of W22 were elevated in both Phase III and Phase IV (Fig. 3A-B). The gradual increase of expression of cluster 4 genes in CML323 aligned well with the increasing need for stress tolerance (response to heat, response to reactive oxygen species, response to toxic substances, response to antibiotics, response to organic nitrogen compound; Fig. S2D). Cluster 4 genes in W22 were mainly involved in growth, immune response, and nutrient transport according to GO analysis (Fig. S3D).

In summary, the results reveal major physiological and biochemical changes occurring in the third stalk node of maize from Phase I to Phase IV. Furthermore, these changes exhibit similarities between both the strong and weak stalk lines. We hypothesize that variations in stalk strength arise from the differential expression of genes associated with significant physiological and biochemical processes that could not be perceived by merely clustering.

### Differentially expressed genes in the two maize lines

**Fig. 4:**
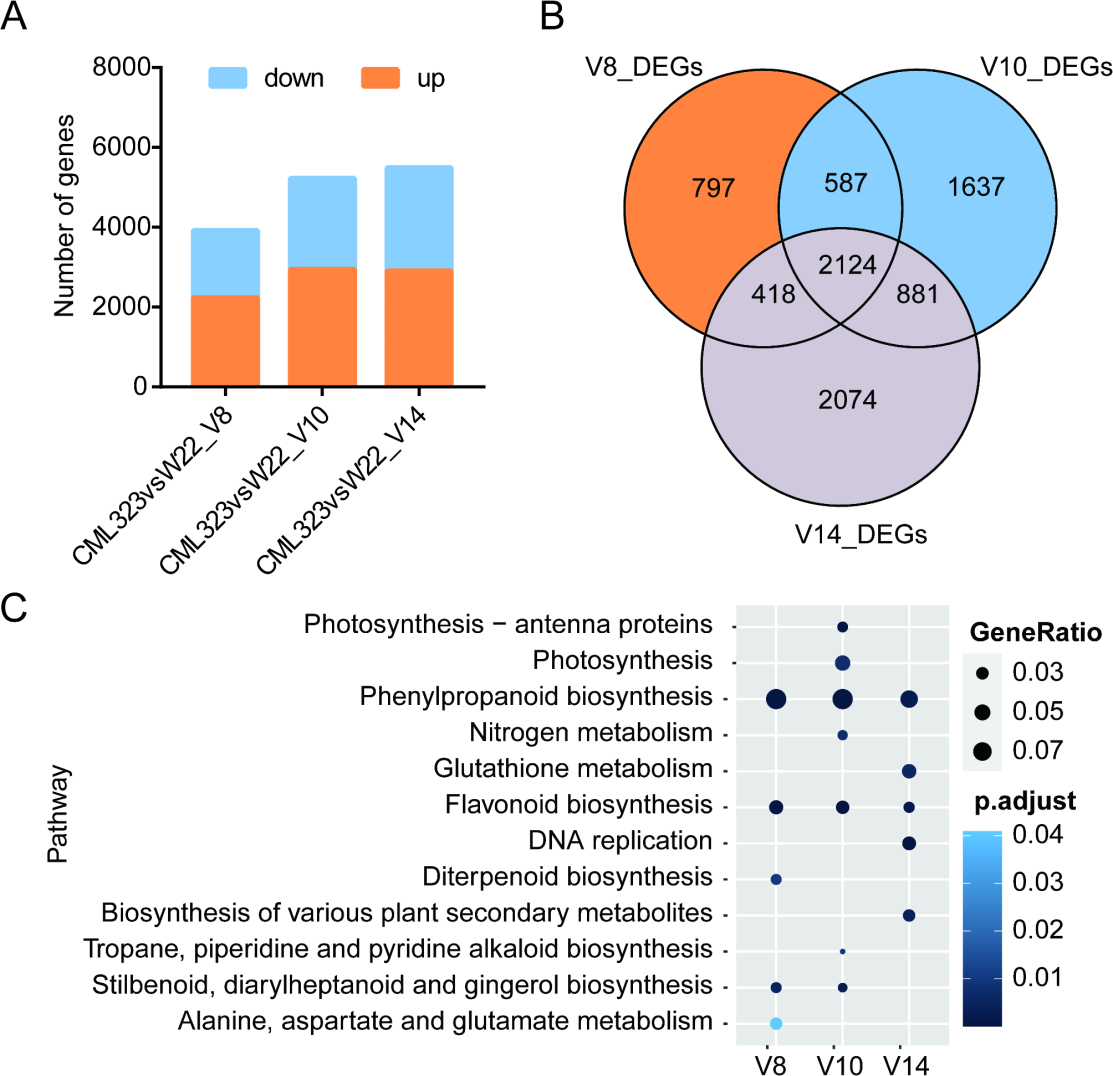
Differential expression analysed between CML323 and W22. (**A**) Number of DEGs between CML323 and W22 in these representative stages. Down and up, down-regulated and up-regulated in CML323 compared with W22. (**B**) The Venn diagram shows the overlap of DEGs of these 3 stages. (**C**) KEGG pathway enrichment analysis of DEGs

In order to understand what genes may participate in the regulation of stalk strength, we chose to analyze differential expression of genes between CML323 and W22 at V8, V10, and V14 (representing Phases I, II, and III). Genes were considered differentially expressed when the P-adjusted value was ≤ 0.05 and the absolute fold change was > 2 between two lines at the same stage [41]. There were 3926 significant DEGs found at V8, 5229 at V10, and 5497 at V14. Among these, 2124 were differentially expressed at all the 3 stages (Fig. 4B). KEGG enrichment analysis of the DEGs at V8 stage revealed that the two lines had significant differences in defense/fortification pathways including phenylpropanoid biosynthesis and flavonoid biosynthesis. These differences remained significant at V10 and V14 stages, and are likely the major factors causing the differences in stalk strength between the two maize lines. At the V10 stage 5229 differentially expressed genes were identified, and KEGG enrichment analysis found that enriched pathways included photosynthesis and nitrogen metabolism, in addition to phenylpropanoid and flavonoid biosynthesis (Fig. 4C). Among these, 22 of the 45 DEGs in the phenylpropanoid biosynthesis pathway at the V10 stage were found to belong to peroxidase gene family, which plays a role as key enzymes in lignin synthesis (Table S5). Polymer of phenylpropanoid (e.g., lignin) are required for mechanical support of plant growth and facilitate long-distance transport of water and nutrients [42, 43], and sufficient lignin deposition and modification of the cell wall are required for maintaining plant tissue mechanical strength [44]. Gene differential expression between the two lines was mainly associated with cell wall deposition and strengthening at V8 and V10 stages. At the V14 stage, 5497 differentially expressed genes were identified, and KEGG enrichment analysis of them revealed additional differences between the two lines including biosynthesis of various plant secondary metabolites, glutathione metabolism, and DNA replication (Fig. 4C).

### Identification of differentially expressed transcription factors

**Fig. 5:**
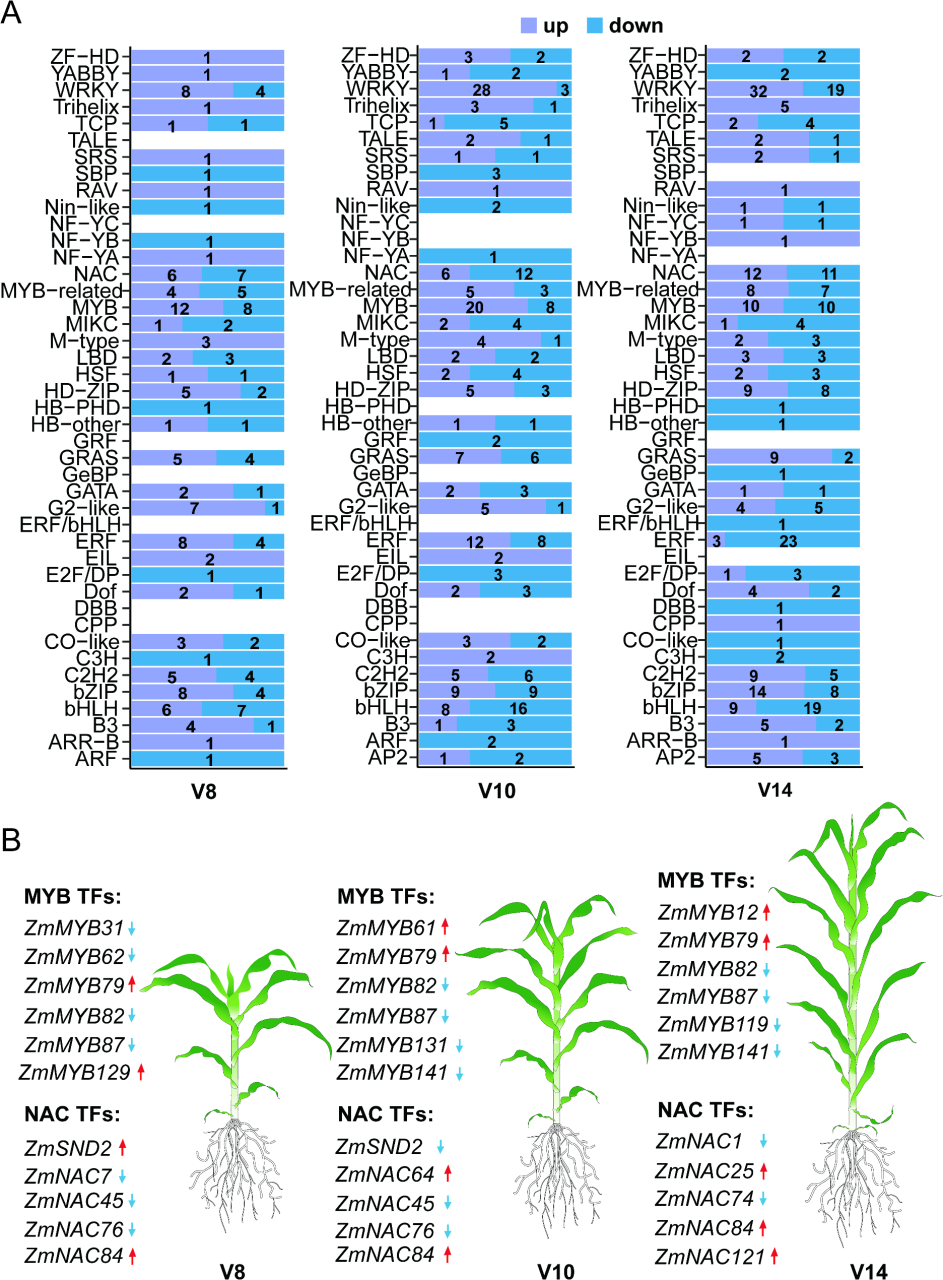
Differentially expressed TFs between CML323 and W22. (**A**) Overview of transcription factors (TFs, ) in the V8, V10, V14 stage. Down and up, purple indicate that the gene is upregulated in CML323, while blue arrows indicate that it is downregulated. (**B**) Top Differentially expressed in NAC and MYB transcription factors. Red arrows indicate that the gene is upregulated in CML323, while blue arrows indicate that it is downregulated

Maize stalk strength is derived mainly from cell wall fortification, and transcription factors are known to play important roles in cell wall regulation [45]. To this end, we investigated TF genes among the differentially expressed genes. There were 174 differentially expressed TFs (belonging to 36 families) at V8, 268 differentially expressed TFs (belonging to 36 families) at V10, and 304 differentially expressed TFs (belonging to 39 families) at V14 (Fig. 5A, Table S4). TF families MYB, WRKY, NAC, bHLH, and ERF contained the large number (top 5) of differentially expressed TFs. Figure 5B shows the top significantly differentially expressed MYB and NAC family TFs. These DEGs demonstrate the different growth regulation capacities/emphases of the two maize lines at these growth stages, and these differences might cumulatively result in the differences in the eventual stalk strength. These differentially expressed TFs deserve further validation in future studies.

### WGCNA identifies functional modules and hub genes associated with stalk strength

**Fig. 6:**
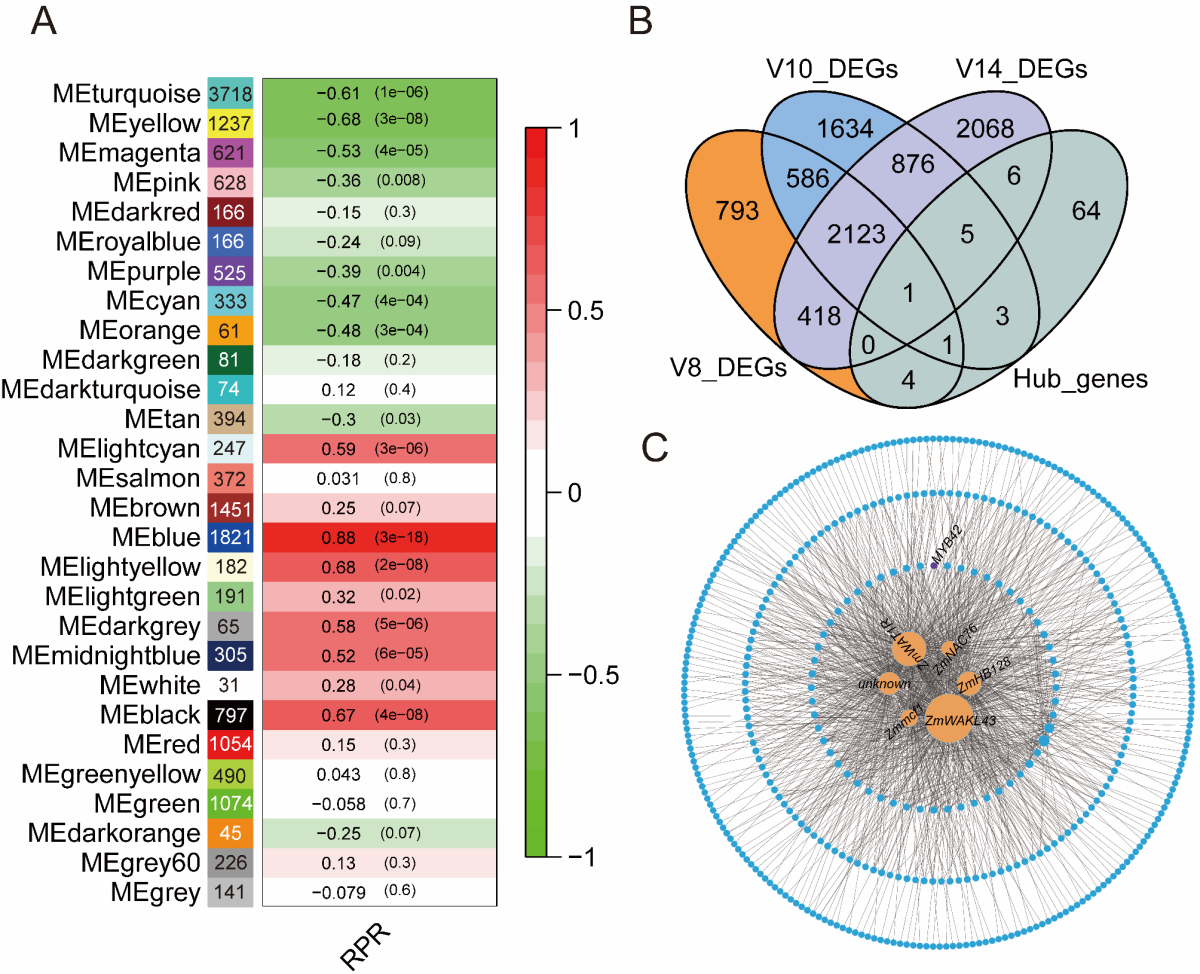
Co-expression network analysis of the dynamically expressed genes and identification of critical genes predicting stalk strength formation. (**A**) Module-phenotype association. Each row represents a colored module, with the number of genes displayed within the colored box of each module. The correlation coefficient between each module and RPR is indicated in red for positive correlations (ranging from 0 to 1) and in green for negative correlations (also ranging from 0 to 1). (**B**) The Venn diagram shows the overlap of DEGs and hub genes. (**C**) Interactions between hub genes within the blue module. Six hub genes are shown as orange circles. *MYB42* is shown as violet circle. RPR, rind penetrometer resistance

To further examine the relationship between the dynamic pattern of maize stalk strength growth and the transcriptional regulatory network, we performed weighted gene co-expression network analysis (WGCNA) based on the correlation between gene modules and the measured stalk strength data. Scale independence and average connectivity were first calculated at different thresholds (Fig. S4). A soft threshold of 8 was chosen to categorize co-expression modules. A cluster dendrogram was created based on the dissimilarity of the topological overlap matrix, and a total of 28 modules consisted of dendrites (Fig. S5A). The module eigengene (ME), which represented the expression level of all genes in the module, was utilized to identify significant correlations between modules and maize stalk strength data. Our analysis focused on the blue module, which contained 1821 genes and had the most significant correlation to RPR (*r* = 0.88; *P* = 3 × 10^− 18^) (Fig. 6A). To verify the relationship between the genes of blue module and the stalk strength, we constructed a correlation scatter plot between the expression of the 1821 genes in the blue module and RPR. Gene expression in the blue module was significantly correlated with RPR (*r* = 0.92; *P* = 1 × 10^− 200^) (Fig. S5B).

To mine key regulatory genes contained within the blue module, a total of 84 hub genes were identified using a screening criterion of key module membership values (kME > 0.8). These 84 genes were then intersected with the DEGs to identify novel key regulators of stalk development. A total of twenty genes were found to be DEGs of the 3 representative stages (Fig. 6B). The co-expression network of these twenty genes in the blue module was constructed by cytoscape using a weight cutoff (> 0.4), in which 6 hub genes showed more connections, including wall-associated kinase-like gene (*ZmWAKL43*/*Zm00001d008468*), WAT1-related protein (*ZmWAT1R*/*Zm00001d045572*), homeobox transcription factor (*ZmHB128*/*Zm00001d021268*), genes with unknown function (*Zm00001d039338*), mitochondrial carrier family protein1 (*Zmmcf1*/*Zm00001d016299*), and NAC transcription factor (*ZmNAC76*/*Zm00001d003626*) (Fig. 6C). We propose that these 6 genes play critical roles in maize stalk development, thereby affecting stalk strength of mature stalks.

### Validation of RNA‑seq data by qRT‑PCR

**Fig. 7:**
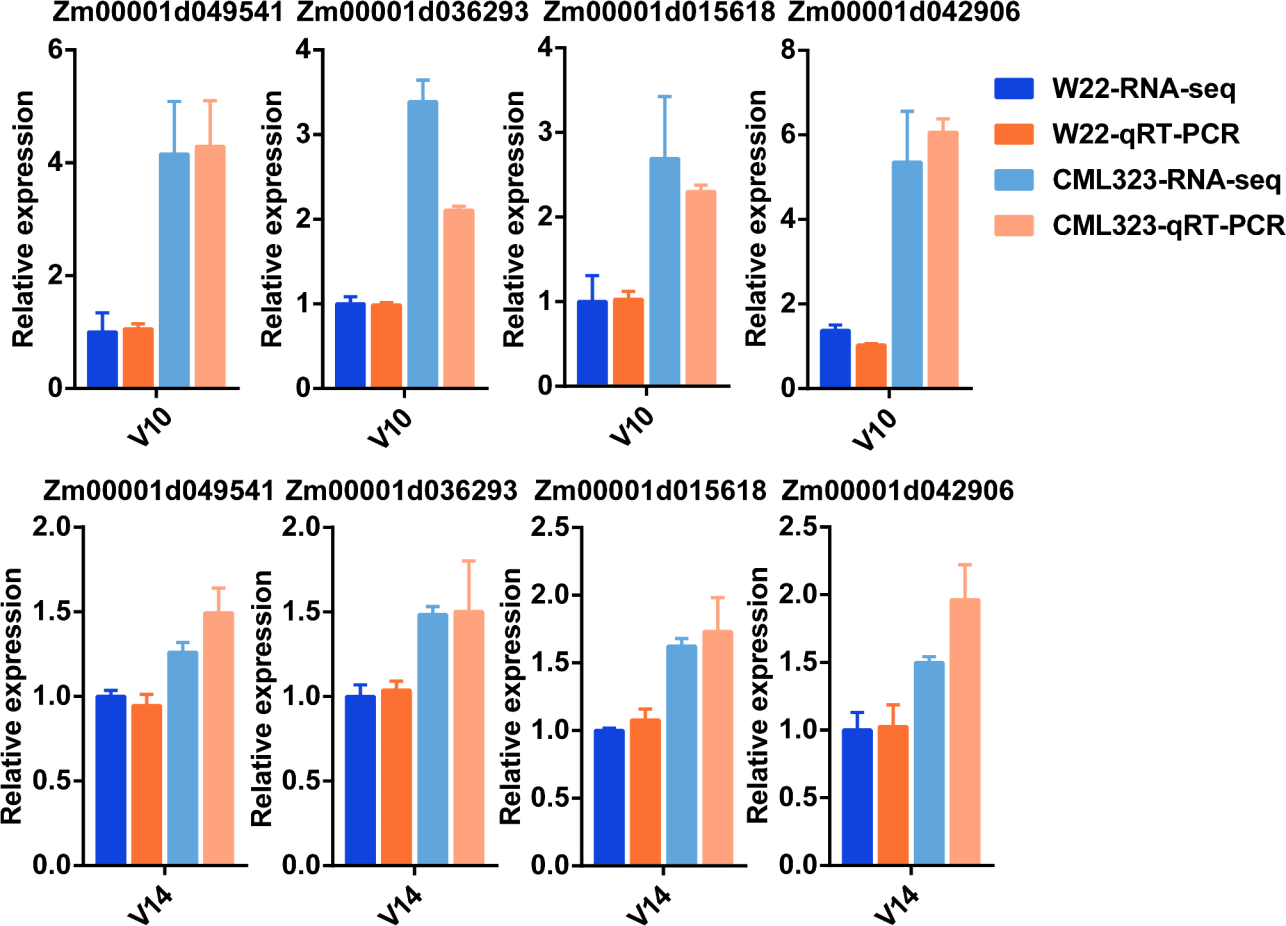
Validation of differentially expressed genes (DEGs) by qRT-PCR. Four DEGs were up-regulated in CML323 compared with W22 at V10 and V14 stages. The relative expression level of each gene was expressed as the fold change in RNA-seq data and qRT-PCR data, setting values of W22 as 1. Error bars represent the standard deviation (*n* = 3)

To validate the accuracy of RNA-seq, we performed qRT‑PCR analysis using the same RNA samples for RNA-seq analysis. DEGs were enriched in the phenylpropanoid biosynthesis pathway (Fig. 4C). Among them, *Zm00001d049541* (*bm3*) mutation was shown to result in reduced lignin content in the stalks, thus increased susceptibility to several diseases [46]; overexpression of *ZmCCoAOMT2* increased lignin content and quantitative resistance to a wide range of pathogens [47]; *ZmCAD2* mutation resulted in reduced lignin content and altered lignin composition [48]; double mutants of *AtLac4* and *AtLac17* resulted in severe defects in lignification of fibers and vessels between stem bundles, whereas triple mutants of *AtLac4*, *AtLac11*, and *Atlac17* resulted in severe growth defects and almost complete loss of lignin in stems and roots [49, 50]; and *Zm00001d042906* (*LAC4*) is found to affect lignin synthesis in cob cells, thereby regulating cob length in maize [51]. The expression patterns of all four DEGs in the qRT-PCR analysis were similar to those in the RNA-seq data (Fig. 7), indicating high reliability of the RNA-seq data.

## Discussion

The transcriptional dynamics during stalk development in maize from the young stem stage to maturity was unclear, and expression of certain genes at critical stages of development may play an important role in determining stalk strength. We constructed a transcriptome time series of the 3rd internode stalk from V7 to silking stage to examine the transcriptional dynamics of maize development. In comparison to previous transcriptomics studies, our study yielded a more comprehensive set of genes associated with these developmental stages. This study is the first complete RNA-seq dataset characterizing a specific stem segment in maize from V7 to silking stage.

Temporal transcriptome data from the two inbred lines implied the existence of four developmental phases of maize stalk development: early growth (Phase I), early elongation (Phase II), late elongation (Phase III), and maturation (Phase IV; Fig. 2). We identified 16,496 dynamically expressed genes and categorized them into nine clusters. These temporal clusters of genes may provide useful information for future studies to look for critical genes that affect traits of the stalk (including the stalk strength) at various stages of stalk development.

Gene differential expression results suggest that development stages V8-V10 are critical for stalk strength formation. For example, the four DEGs selected for qPCR validation were all upregulated in CML323 at V10 (Fig. 7). On the other hand, differential expression of transcription factors may have important consequences. The ERF family transcription factors regulate secondary cell wall synthesis. For example, *ERF139* modulates the process of vascular bundle expansion, stimulating G-type lignin accumulation while inhibiting H-type lignin [52]. The interaction between *GhERF108* and *GhARF7-1* or *GhARF7-2* activates *GhMYBL1*, which then triggers the activation process of fiber secondary cell wall biosynthesis [53]. WRKY TFs regulate plant growth, development, and height in a variety of species, and *ZmWRKY92* binds to jasmonic acid synthesis-related genes to regulate stalk cell size and influence plant height [54]. *AtWRKY12* is expressed in the pith and cortex, where it inhibits secondary wall formation by directly repressing secondary wall master regulators such as *NST2* and causes an ectopic secondary wall in the pith [55]. Basic Helix-Loop-Helix (bHLH) TFs are involved in the regulation of plant growth and development, including morphogenesis [56], promoting cell expansion and lignin deposition [57], and maize internode elongation [58]. MYB and NAC families play important roles in the TF network regulating secondary wall synthesis. A conserved NAC-MYB regulatory network for secondary cell wall development in plants dynamically regulates cellulose and lignin contents in stalks [1, 59–61]. *Zm00001d053220* (*ZmMYB42*) reduces lignin synthesis by repressing the expression of lignin biosynthesis genes such as *ZmCOMT1*, and *Zm4CL2* [62]. *ZmMYB69* can inhibit lignin biosynthesis through the activation of *ZmMYB31* and *ZmMYB42* expression [63]. *ZmNST3* has been demonstrated to activate the expression of genes associated with secondary cell wall cellulose biosynthesis by binding to the promoters of *CESA5* and Dynamin-Related Proteins2A (DRP2A) [64].

The plant cell wall is a natural nanoscale network structure composed mainly of polysaccharide polymers such as cellulose, hemicellulose, lignin, and pectin [45]. Among this, Lignin is the most significant product of the phenylpropanoid pathway [65]. Based on the results of KEGG enrichment analysis, the two maize lines had significantly different expression profiles in the phenylpropanoid biosynthesis pathway and the flavonoid biosynthesis pathway at V8, V10 and V14 stages (Fig. 4C). These pathways contain a variety of enzymes involved in lignin synthesis, including Cinnamate 4-hydroxylase (C4H) family, cinnamoyl-CoA reductase (CCR) family, cinnamyl alcohol dehydrogenase (CAD) family, peroxidases and laccases. Among these, *ZmC4H* (*Zm00001d012510*), *ZmCCRs* (*Zm00001d045101* and *Zm00001d050417*), *ZmCAD* (*Zm00001d045043*), *ZmPOD* (*Zm00001d040364*) and *ZmLAC* (*Zm00001d012408*) were expressed at significantly higher levels in CML323 than in W22 at least one stage (Tables S6, S7, S8).

Combining WGCNA with stalk strength phenotyping, we identified 28 co-expression modules that were correlated with stalk strength (Fig. 6A). By intersecting of 84 hub genes in blue module with DEGs, a total of twenty genes were identified. Six hub genes (*ZmWAKL43*, *ZmWAT1R*, *ZmHB128*, *unknown*, *Zmmcf1*, and *ZmNAC76*) showed more connections in network (Fig. 6C), which contained a NAC transcription factor *ZmNAC76*. The NAC family plays an important role in the TF network that regulates secondary wall synthesis [66], with *SND1* and *NST1* being the master switches that activate secondary wall biosynthesis in fibers, while *VND6* and *VND7* are responsible for activating secondary wall biosynthesis in vascular [67]. In the co-expression network, *ZmMYB42*, which regulates lignin synthesis, is a putative target gene of *ZmNAC76* (Fig. 6C). We believe that these six hub genes in the co-expression network had significant effect on stalk development through different pathways, as they were differentially expressed in the two maize lines of significantly different stalk strengths. However, the exact roles of six hub genes in maize stalk development remain to be further explored.

## Conclusions

In this study, we report a temporal resolution transcriptome map and discussed potential regulatory genes involved in maize stalk development and strength formation. The typical developmental stages were harvested to analyze the changing of gene expression to systematically elucidate the specific events that occur during the four phases of the maize stalk transition from early development to maturity. The DEGs were enriched in phenylpropanoid and flavonoid biosynthesis pathways during the V8, V10, and V14 stages, which may be related to stalk strength formation. WGCNA and differential expression analysis predicted key genes that may have profound impact on the maize stalk strength trait. These results facilitate future investigations into maize stalk development and stalk strength, laying a foundation for such research.

## Electronic supplementary material

Below is the link to the electronic supplementary material.

Supplementary Material 1

Supplementary Material 2

Supplementary Material 3

Supplementary Material 4

Supplementary Material 5

Supplementary Material 6

Supplementary Material 7

Supplementary Material 8

Supplementary Material 9

Supplementary Material 10

Supplementary Material 11

Supplementary Material 12

Supplementary Material 13

Supplementary Material 14

Supplementary Material 15

Supplementary Material 16

## Data Availability

The RNA sequence data can be found in the National Genomics Data Center (NGDC) database with the accession number CRA020541. All data generated or analyzed during this study are included in this published article and its supplementary information files.

## Abbreviations

RPR: Rind penetrometer resistance
NSS: Non-Stiff Stalk
SS: Stiff Stalk
WGCNA: Weighted gene co-expression network analysis
V7: Seventh leaf stage
Vn: Nth leaf stage
VT: Tasseling stage
R1: Silking stage
R2: Blister stage
TPM: Transcripts per million
PCA: Principal component analysis
CV: Coefficient of variation
Csl: Cellulose synthase-like
CCR: Cinnamoyl-CoA reductase
WAK/WAKL: Wall-associated kinase/wall-associated kinase-like
GO: Gene Ontology
BR: Brassinosteroid
KEGG: Kyoto Encyclopedia of Genes and Genomes
DEGs: Differentially expressed genes
TF: Transcription factor
bHLH: Basic Helix-Loop-Helix
C4H: Cinnamate 4-hydroxylase
CAD: Cinnamyl alcohol dehydrogenase
CesA: Cellulose synthase
COMT: Catechol-Omethyl transferase
4CL: 4-coumarate CoA ligase
CCoAOMT: Caffeoyl-CoA-O methyltransferase
lac: Laccase
kME: Key module membership
