# Supplementary figures and images for: Comparative transcriptome analysis reveals potential regulatory genes involved in the development and strength formation of maize stalks
Source: BMC Plant Biol. 2025 Mar 1;25:272. doi: 10.1186/s12870-025-06276-5 (PMC11871777; doi:10.1186/s12870-025-06276-5)

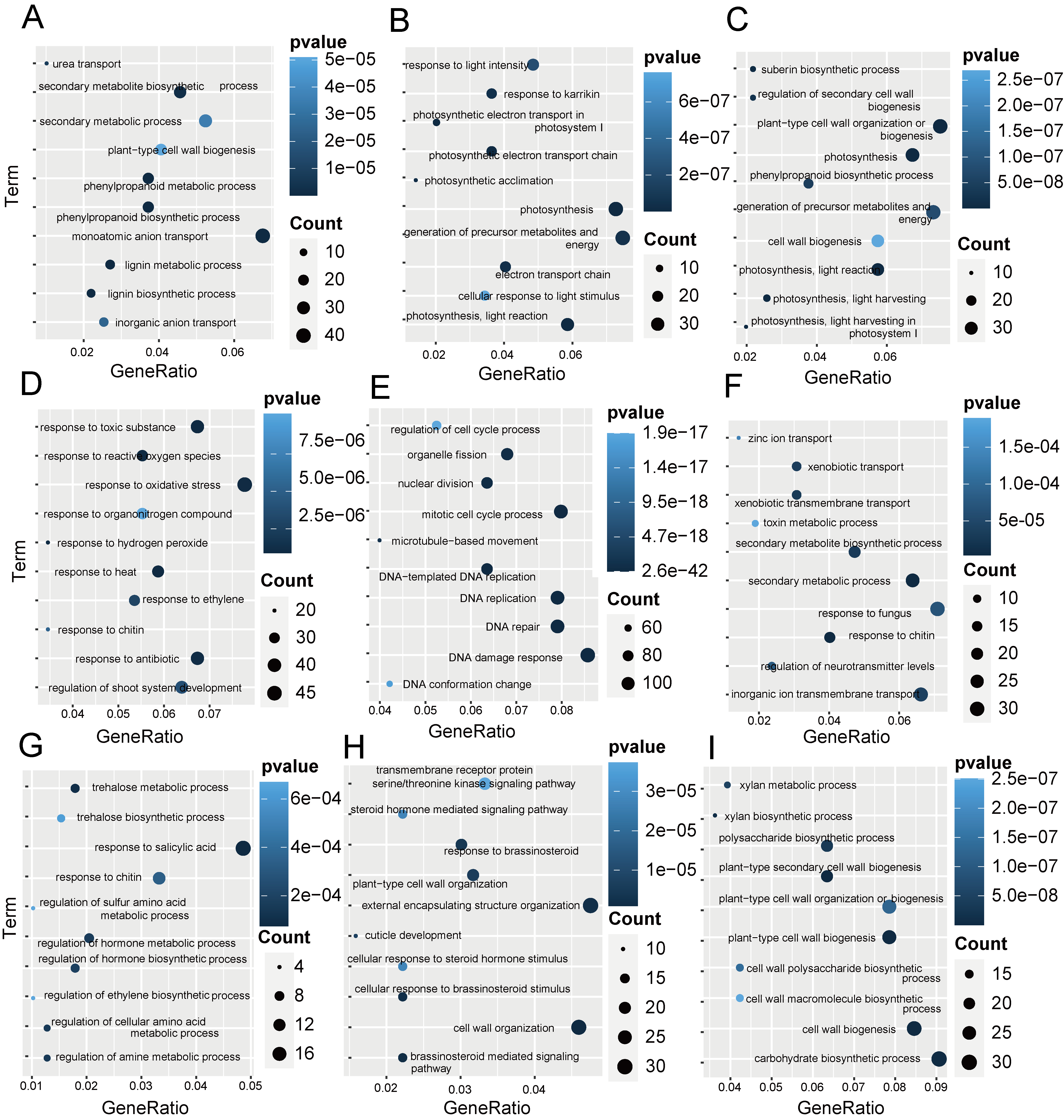

Supplement: Supplementary file 2 — Supplementary Material 2 [file 12870_2025_6276_MOESM2_ESM.tif]

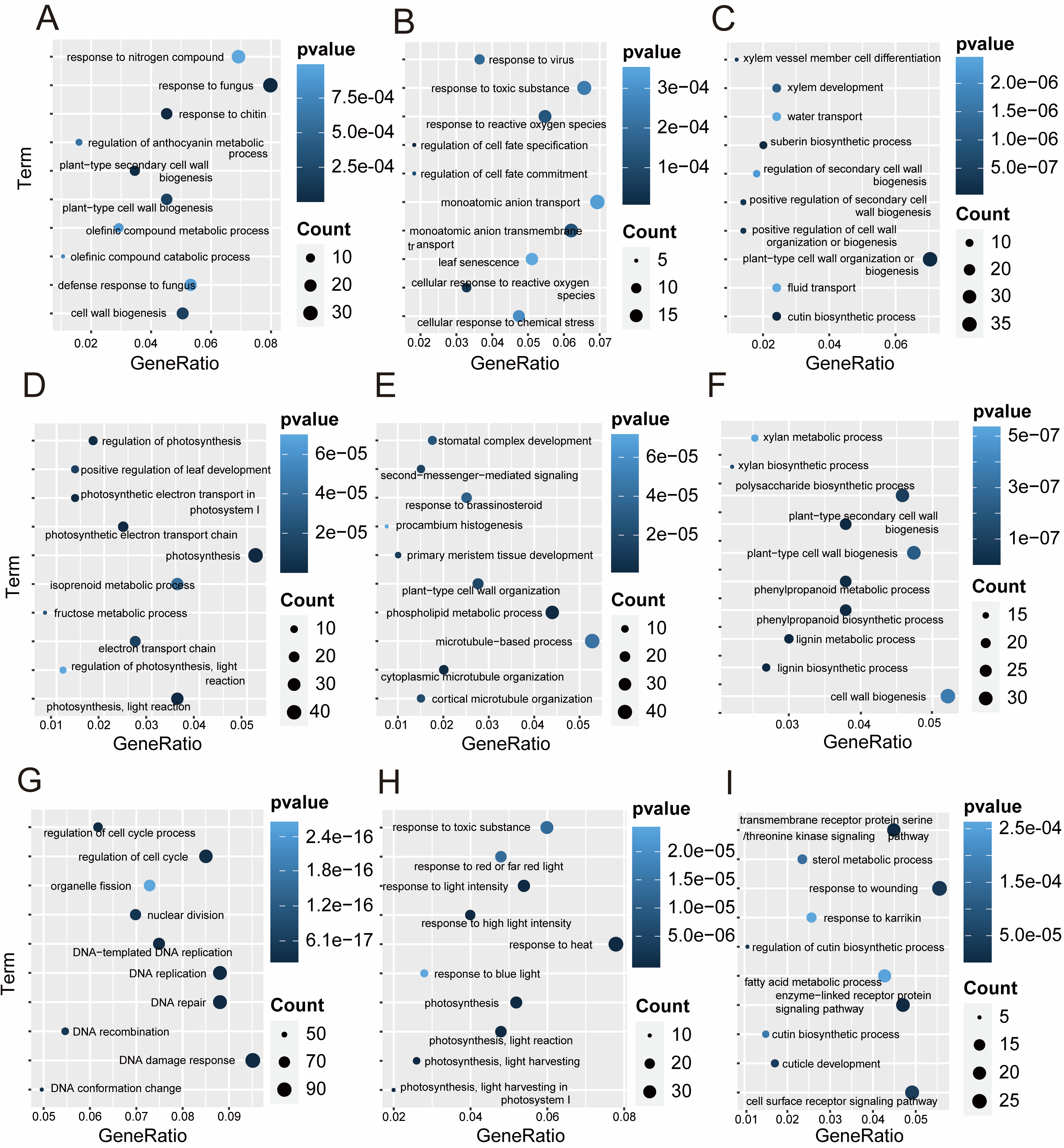

Supplement: Supplementary file 3 — Supplementary Material 3 [file 12870_2025_6276_MOESM3_ESM.tif]

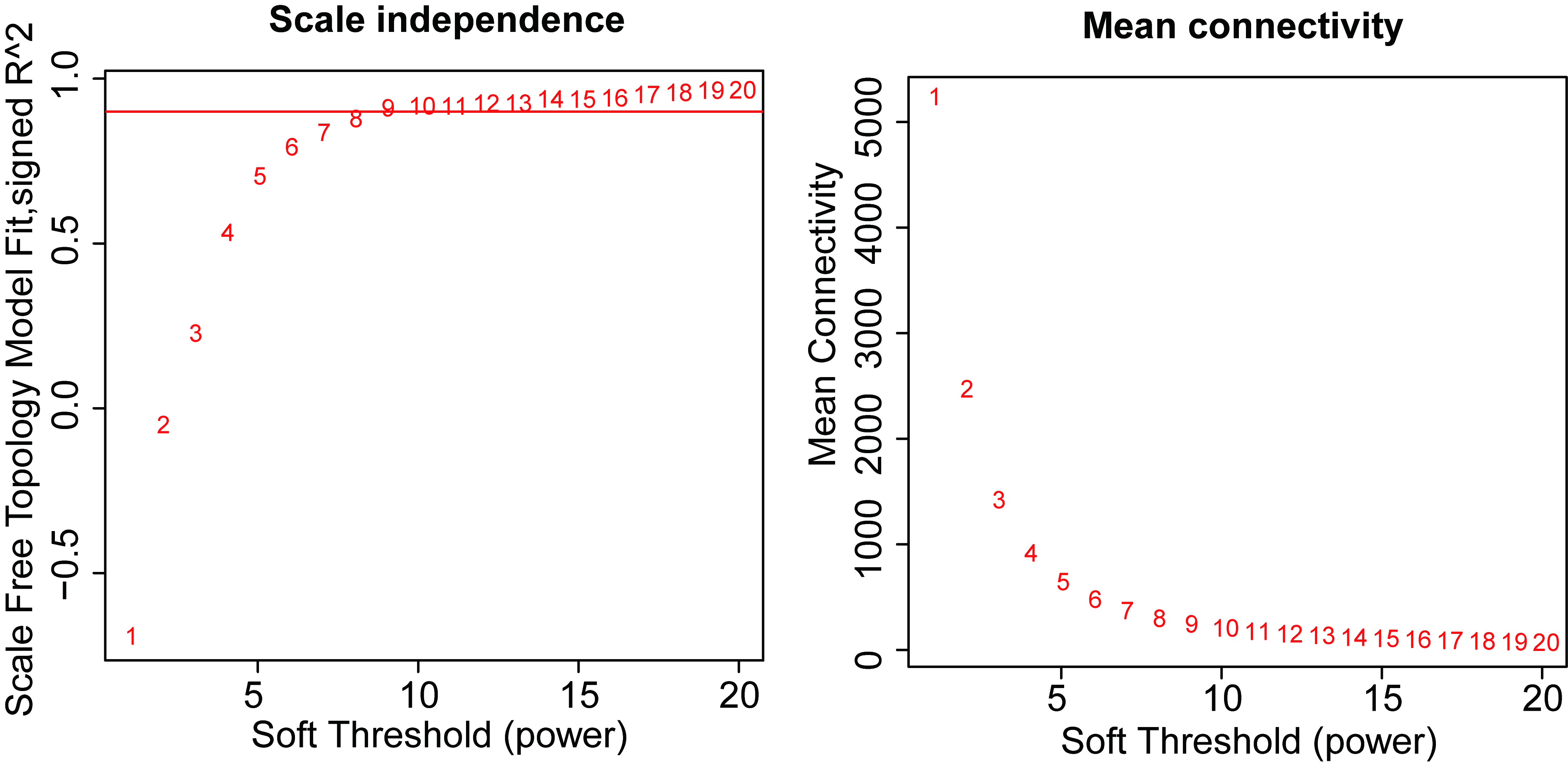

Supplement: Supplementary file 4 — Supplementary Material 4 [file 12870_2025_6276_MOESM4_ESM.tif]

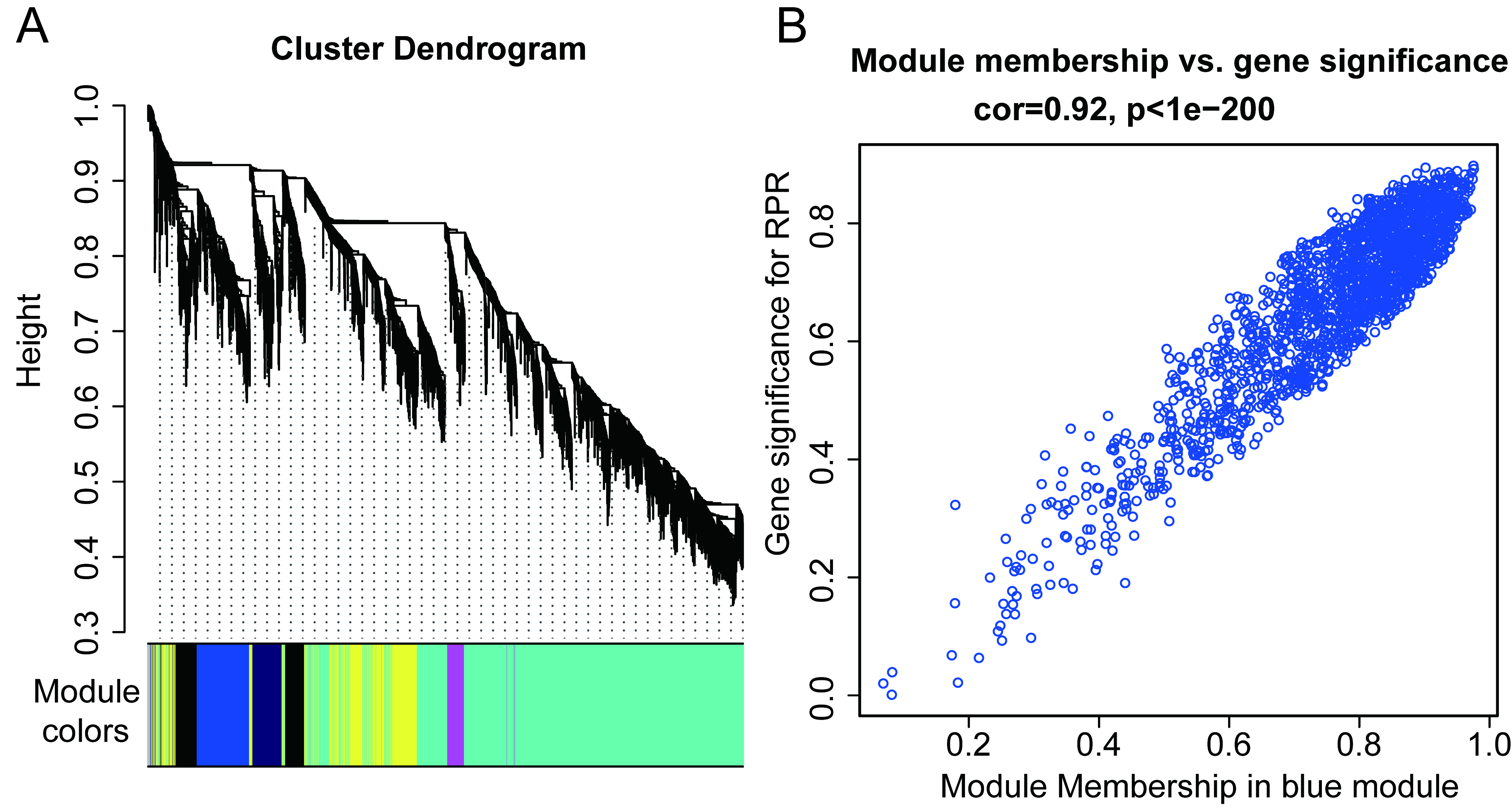

Supplement: Supplementary file 5 — Supplementary Material 5 [file 12870_2025_6276_MOESM5_ESM.tif]
