# Supplementary material for: Comparative transcriptome analysis reveals potential regulatory genes involved in the development and strength formation of maize stalks
Source: BMC Plant Biol. 2025 Mar 1;25:272. doi: 10.1186/s12870-025-06276-5 (PMC11871777; doi:10.1186/s12870-025-06276-5)
Supplement: Supplementary file 6 — Supplementary Material 6 [file 12870_2025_6276_MOESM6_ESM.docx]

**Supplementary material**

**Additional file 1: Figure. S1.** PCA of the transcriptomes. (A) PCA of the transcriptomes of CML323. (B) PCA of the transcriptomes of W22.

**Additional file 2: Figure. S2.** GO functional enrichment analysis of gene expression patterns in CML323. (A-I) Functional category enrichment of the nine fuzzy c-means clusters, Cluster 1 (A), Cluster 2 (B), Cluster 3 (C), Cluster 4 (D), Cluster 5 (E), Cluster 6 (F), Cluster 7 (G), Cluster 8 (H), and Cluster 9 (I).

**Additional file 3: Figure. S3.** GO functional enrichment analysis of gene expression patterns in W22. (A-I) Functional category enrichment of the nine fuzzy c-means clusters, namely Cluster 1 (A), Cluster 2 (B), Cluster 3 (C), Cluster 4 (D), Cluster 5 (E), Cluster 6 (F), Cluster 7 (G), Cluster 8 (H), and Cluster 9 (I).

**Additional file 4: Figure. S4.** Scale independence and mean connectivity of the network at different soft-threshold powers. The left panel displays the correlation of the soft-threshold with the scale-free fit index. The right panel displays the influence of soft-threshold power on mean connectivity.

**Additional file 5: Figure. S5.** Module identification and the correlations between RPR and critical module genes. (A) Hierarchical cluster tree showing co-expression modules identified by WGCNA. The major tree branches constitute 28 modules labeled with different colors. (B) Scatter plot showing the correlations between genes in the blue module and RPR value.

**Additional file 6: Table S1.** Summary of RNA-Seq data.

**Additional file 7: Table S2.** Gene clusters of maize stalks in CML323.

**Additional file 8: Table S3.** Gene clusters of maize stalks in W22.

**Additional file 9: Table S4.** List of differentially expressed TFs at 3 stages.

**Additional file 10: Table S5.** List of DEGs in the phenylpropanoid biosynthesis at the V10 stage.

**Additional file 11: Table S6.** List of DEGs between CML323 and W22 at V8 stage.

**Additional file 12: Table S7.** List of DEGs between CML323 and W22 at V10 stage.

**Additional file 13: Table S8.** List of DEGs between CML323 and W22 at V14 stage.

**Additional file 14: Table S9.** The interactions networks within the blue module.

**Additional file 15: Table S10.** List of PCR primers used in this study.
